# Performance Evaluation of an IoT Sensor Node for Health Monitoring of Artwork and Ancient Wooden Structures

**DOI:** 10.3390/s22249794

**Published:** 2022-12-13

**Authors:** Ada Fort, Elia Landi, Marco Mugnaini, Lorenzo Parri, Valerio Vignoli

**Affiliations:** Department of Information Engineering and Mathematical Sciences, University of Siena, 53100 Siena, Italy

**Keywords:** environmental monitoring, smart sensors, IoT, sensor networks, artwork monitoring, vibration monitoring, supply chain visibility

## Abstract

In this paper, an IoT sensor node, based on smart Bluetooth low energy (BLE), for the health monitoring of artworks and large wooden structures is presented. The measurements from sensors on board the node are collected in real-time and sent to a remote gateway. The sensor node allows for the monitoring of environmental parameters, in particular, temperature and humidity, with accurate and robust integrated sensors. The developed node also embeds an accelerometer, which also allows other mechanical quantities (such as tilt) to be derived. This feature can be exploited to perform structural monitoring, exploiting the processing of data history to detect permanent displacements or deformations. The node is triggered by acceleration transients; therefore, it can also generate alarms related to shocks. This feature is crucial, for instance, in the case of transportation. The developed device is low-cost and has very good performance in terms of power consumption and compactness. A reliability assessment showed excellent durability, and experimental tests proved very satisfactory robustness against working condition variations. The presented results confirm that the developed device allows for the realization of pervasive monitoring systems, in the context of the IoT paradigm, with sensor nodes devoted to the monitoring of each artwork present in a museum or in a church.

## 1. Introduction

Museums and churches are the places where most artworks and artifacts are stored and put on display to the public. In these sites, the degradation of artworks and artifacts is mostly due to their exposition to human factors and environmental and climatic variations. For instance, humidity and temperature variations affect not only the presence of microbiological organisms, which can deteriorate the visual appearance of an artifact but also heavily degrade its mechanical strength [1,2]. For this reason, it is of the utmost importance to monitor the environmental conditions near the individual artwork [3], as well as the vibrations and deformations transmitted to it.

Many distributed monitoring systems aimed at the environmental monitoring of the microclimate surrounding the artwork already exist in the literature [4], as well as studies related to the optimization of heating, ventilation, and air conditioning (HVAC) in museums [5]. Microclimatic monitoring systems are also used to predict the presence and the evolution of mold and fungal species inside the structure and on the surfaces of the artworks. Mold, in fact, can grow according to precise combinations of humidity, temperature, and substrate [6,7].

Considering an artwork and its surrounding environment, especially in ancient churches, where wood is massively used, it is important to monitor not only the climatic conditions but also the movement of timber structures near the artwork or of the artwork itself. Variations in humidity and temperature, in fact, can cause variations in the moisture content of the wood, which can also cause large deformations in the structures [7,8,9].

As already said, it is important to monitor the vibrations which are transmitted from the hosting infrastructure to the artwork itself. In this respect, several works treat this subject and introduce methods to dampen the transmission of vibrations [10,11].

Another aspect related to the conservation of cultural heritage is the safety of artworks during transport. Additionally, in this case, vibrations are the biggest problem since they can cause permanent damage. During transportation, in fact, rapid mechanical loading can occur, and especially during the loading and unloading phases, out-of-plane vibrations can happen. These can be dangerous when the frequency is near the resonant frequency of the artifact itself [12]. Monitoring techniques rely on laser displacement sensors [13] and accelerometers [14]. In particular, the work reported in [14] shows that the biggest part of the vibrations, which can occur during transportation, can fall in the 0–5 Hz range with minor amplitudes in the 50–500 Hz range. Nowadays, several companies offer products and services related to supply chain visibility; however, the services they provide are usually related to the location of the goods with temperature monitoring during transport, leaving out the monitoring of shocks and vibrations suffered by objects.

Sensor nodes for artwork monitoring have already been implemented in the past. These devices aimed at monitoring the local ambient parameters and relied on wireless communication to send data to the user [15,16,17]. In particular, Bluetooth low energy (BLE) has been widely used in modern sensor nodes for artwork monitoring [18].

The integration of vibration sensors in nodes for artwork monitoring has been discussed in [19]; however, in this work, the adopted accelerometer is not a precision sensor and has been used only to detect shock. Moreover, the sensor nodes presented in the aforementioned works have non-negligible sizes, which makes it difficult to embed them in the artwork. 

In this context, the authors present a sensor node in [20], which is able to monitor climatic conditions such as temperature, humidity, and pressure, but also vibrations, shocks, and tilt. The node can be used to determine the health of artworks in museums or churches, as well as the health of ancient timber structures and wooden artworks, such as lecterns and pulpits. The small size of the proposed node, its low cost, and it’s very low power consumption allow for the realization of pervasive monitoring systems, where sensor nodes are devoted to the monitoring of each artwork present in a museum or in a church or are deployed to monitor different zones of large timber structures.

The developed node is based on an STM32 microcontroller and embeds BLE technology. Nodes can be connected in a sensor network exploiting a BLE gateway, and the data, which are transmitted to a remote server, are available to the final user. The node has an optimized design in terms of size, ease of deployment, cost, and power consumption and allows for a minimally invasive placement. It can be used in different application scenarios, from microclimate to artwork health monitoring, but also for safety applications, due to the presence of the vibration sensor. 

Moreover, nodes are designed to be used both in fixed installations and during transportation since they are powered by a battery. The low-power design allows the battery duration to be extended up to 1 year with a CR2477N battery (3 V, 950 mAh).

Concerning this aspect, several devices have been studied in terms of power consumption reduction or small dimensions for indoor or industrial applications. Nevertheless, the reliability aspects connected to these applications have to be deeply investigated when considering different missions and exploitation scenarios. Presently, there may happen to be a device that operates with different duty cycles according to the specific application or to the peculiar working conditions or to have devices which undergo different mission phases. The reliability and robustness of the proposed sensor node, in terms of durability and measurement performance in different working conditions, were analyzed by means of reliability studies and experimental tests.

This paper is organized as follows: in Section 2, the monitoring system is described in detail. In Section 3, the reliability and availability studies are presented together with the obtained results. In Section 4, the tests in different working conditions are described, and the obtained results are presented and discussed. Finally, the conclusions are drawn in Section 5.

## 2. Sensor Node and Network Description

In this section, the structure of the monitoring system and the proposed sensor node will be described. Two different data collection scenarios related to the context in which the device is used will be provided. 

### 2.1. Sensor Node Realization

**Figure 1:**
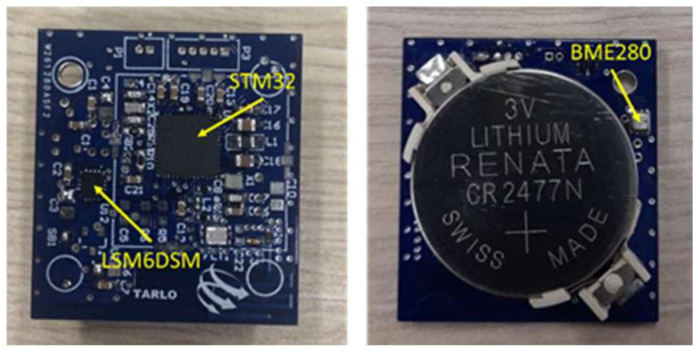
Representation of the hardware of the developed sensor node.

The proposed sensor node is designed to acquire environmental parameters as well as vibration and tilting signals, process the data, and exploit a BLE communication interface to send the obtained information to a remote gateway. The device was designed by exploiting an STM32 microcontroller that embeds the BLE communication interface. The sensor node hosts digital sensors that are interfaced with the microcontroller SPI interfaces. The developed node can be seen in Figure 1.

The microcontroller acquires data at regular intervals from the sensors (10 s), and it advertises BLE packets every two seconds, while for the rest of the time, it is maintained in low power mode to save battery energy. The node has been designed to be powered by a replaceable primary Lithium battery model CR2477N with a rated capacity of 950 mAh. Below the sensing circuits are described in more detail.

#### 2.1.1. Environmental Parameters Measurement

The environmental parameters acquired by the node are temperature, relative humidity, and pressure by means of a digital sensor Bosch BME280 which communicates with the STM32 microcontroller via an SPI interface. The sensor was selected due to its low power consumption (3.6 μA) and its satisfactory metrological specifications. In particular, the temperature sensor operates in the range of −40 °C–+85 °C, with a resolution of 0.01 °C and an accuracy of ±0.5 °C, whereas the humidity sensor range is 0% RH—100% RH, with a resolution of 0.008% RH and an accuracy of ±3% RH. Regarding the atmospheric pressure sensors, it works in the range 300 hPa–1100 hPa with a resolution of 0.18 hPa, and an accuracy of ±1 hPa.

#### 2.1.2. Vibration and Tilting Measurements

Vibrations and tilting are measured by an ST LSM6DSM MEMS IMU, which is interfaced with the processor through an SPI interface. This unit was characterized by the authors in some previous works [21] using a test bench composed of a vibrating source and a reference accelerometer.

Here, the acceleration magnitude as a function of frequency was measured and compared with the one provided by the piezoelectric reference accelerometer (B&K 4326A). Measurements show that the relative error of the measured magnitude is lower than 10% up to 700 Hz [22].

The IMU acceleration full scale was set to ±2 g (1 g = 9.81 m/s^2^) with a resolution of 1 mg, in this setting, with an accuracy of ±1 mg. The angles needed to recover the movement of the sensor node in the space were evaluated from the acceleration. In fact, to extend the battery life, the gyroscope present in the IMU was not used. The current consumption of the IMU sensor using only the accelerometer is 9 μA, while when using the gyroscope it is 0.29 mA due to the needed activity to drive the seismic masses in the MEMS. 

As already stated, since the gyroscope was not used, the information about the tilt was derived on the server side based on the accelerometer output and according to the following equation, providing the assumption that the only static acceleration is gravity:

(1) $$\phi_{i}=\frac{360}{2\pi }acos\left(\frac{a_{i}}{\sqrt{a_{x}{}^{2}+\;a_{y}{}^{2}+a_{z}{}^{2}}}\right)$$

where $\phi_{i}$ is the angle of inclination of the *i*-axis with respect to gravity, and *a_i_* $i\;\in \;\left\{x,y,z\right\}$ are the DC accelerations measured by the triaxial accelerometer 

Applications in fixed installations where the node can be powered by the main power or by large batteries can also exploit the gyroscope to detect the angle with better accuracy.

The LSM6DSM chip is equipped with an accelerometer hardware interrupt generator, which is exploited to wake up the microcontroller in the case of motion or vibration detection. Using this feature, it is possible to acquire and immediately transmit the acceleration data due, for example, to shocks or impacts, which would otherwise be lost.

#### 2.1.3. Battery Life Estimation

To assess the battery life of the proposed device, the current consumption was acquired during the operation of the node, in the presence and in the absence of data transmissions. As previously introduced, in the absence of variations of the position or vibration events (which trigger the accelerometer and generate the hardware interrupt), the node in the IDLE mode transmits at regular intervals when set by default; when a hardware interrupt is generated, the node enters the so-called METERING MODE, in which it transmits the acquired data more frequently with respect to the IDLE mode. In typical applications, the time interval between the transmission of the BLE adverts is set to 10 s in IDLE MODE and at 2 s in the presence of vibrations, i.e., in METERING MODE.

**Figure 2:**
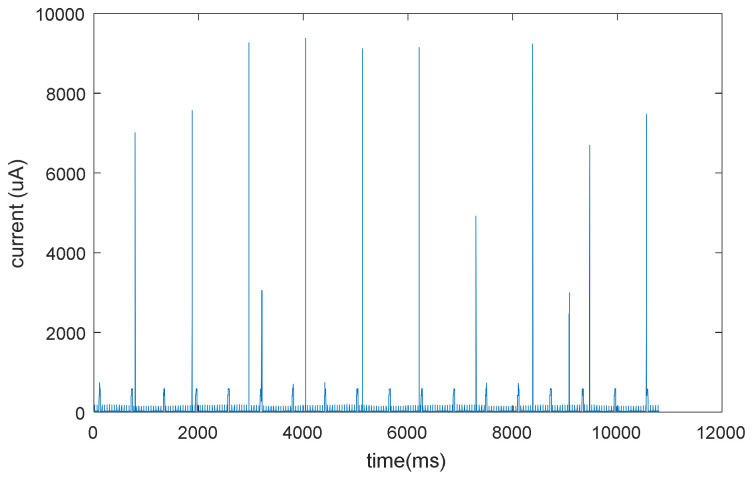
Node current consumption as a function of time, in the presence of vibration-triggered events.

Figure 2 represents the node current consumption as a function of time in the presence of vibration-triggered events. In particular, to obtain these data, the transmission interval in the presence of vibrations and angle variations was set to 1 s to have a worst-case scenario in the evaluation of the node power consumption.

In this condition, the node’s average current consumption is 66.8 µA, and assuming that the node is supplied with a CR2477N battery, the expected battery life is about 1.6 years. 

### 2.2. Sensor Network and User Interface

**Figure 3:**
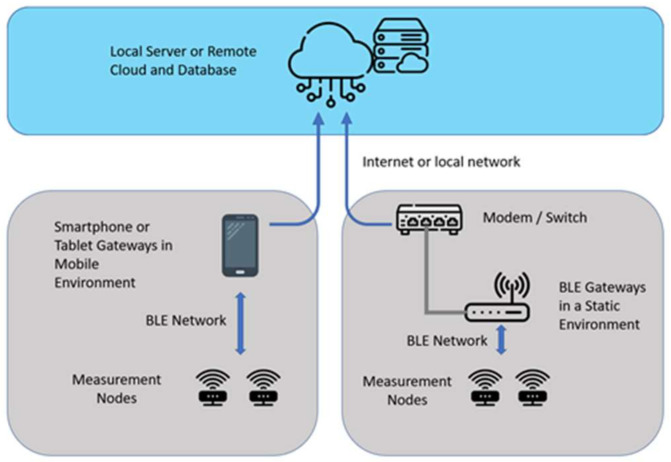
Data collection network scenarios.

As already mentioned, the sensor node data are periodically transmitted as a BLE advertisement message. The advertisement period is 10 s in the absence of vibration and tilting events. The data collection network (Figure 3) was designed for two possible scenarios: a static environment where devices are in a fixed position and one or more gateways can be installed in a mobile environment (such as delivery or transport monitoring) where a smartphone or a tablet can be used as a gateway.

In the static environment, the data transmitted from the BLE nodes can be collected by standard gateways connected to the internet by the building network infrastructure. If two gateways receive the same data from a node, a duplicated packet will be transmitted to the server. 

This fact does not represent an issue since the server is configured to save a maximum number of samples from a node per unit of time (e.g., 1 sample every 30 s can be a good tradeoff for the application). This last rule is not applied to packets related to a change in the accelerometer data; in this case, data are saved on the database even if they are more frequent than the preset sampling time previously mentioned. This policy is used to detect position changes or sudden vibrations, which, as explained in the previous section, are immediately transmitted by the sensor nodes.

In the mobile environment, the same data collection strategy is maintained except for the usage of a mobile phone or a tablet with an application that emulates a gateway. Data are transmitted to the server by the cellular network. This scenario is applicable to the case of artwork transport monitoring, where no fixed gateway can be installed or powered. 

The server is composed of a Nodered-based back-end time-series database (influxdb) and a dashboard based on Grafana. The gateway used for the test was based on a Raspberry Pi and a Nodered backed, and any type of commercial BLE gateway could be used for the application. Data are sent to the remote server by an MQTT protocol exploiting Mosquitto as a broker.

## 3. Reliability and Availability of the Proposed Sensor Node

As previously described, the sensor node architecture can be easily represented as a simple series system that is composed of three well-identified modules. The first module containing a core and transmission unit (MCU + BLE) is able to acquire the signals coming from the sensors and to transmit efficiently to a gateway, the module for tilting and vibration data acquisition (tilting and vibration), and the module containing the sensing unit composed by the humidity, temperature, and pressure sensors (Sensors).

All these units can be combined in a reliability block diagram (RBD) as a series system where the reliability relationship can be expressed by (2):

(2) $$R\left(t\right)=\prod_{i=1}^{n}\;e^{-\lambda_{i}t}=e^{-\overset{n}{\underset{i=1}{\sum }}\lambda_{i}t}$$

**Figure 4:**
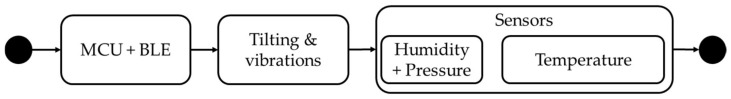
RBD of the sensor node as a series system.

In (2), λ*_i_* represents the individual failure rate of each component comprised in the system. The whole system is, therefore, considered working if and only if all the components are working and their RBD representation is the one in Figure 4.

Once the RBD is set, the mission profiles should be identified, and the corresponding working phase assigned properly. For this device, in particular, two different operating modes were designed and, therefore, considered: the ‘continuous’ and the ‘on-demand’ mode. The first one is intended when the three blocks composing the RBD are always working and continuously acquiring and generating signals. Therefore, it can be considered that the blocks are 100% on duty for the whole mission. A more appropriate way to represent the sensor node behavior in terms of reliability is the on-demand mode since the sensors are used only for a small part of the transmission cycle period.

The environment was tested from the ground benign up to the airborne uninhabited cargo, and the operating temperature was set to 25 °C. The reliability database was selected to be the ANSI VITA 5.0 in order to obtain a less conservative approach with respect to the MIL-HDBK217F. This latter, as a matter of fact, provides results that are generally too conservative with respect to actual commercial applications.

The results of the reliability analysis reported in the following subsections are expressed in terms of MTBF (mean time between failures), where MTBF = $\int_{0}^{\infty }R\left(t\right)dt,$ or $\lambda \;=\frac{1}{MTBF}$, where λ represents the failure rate evaluated in different conditions and working modes.

The failure rate can be expressed even as its correspondent hazard function h(t):

(3) $$h\left(t\right)=\underset{\Delta t\to 0}{\lim}\frac{N_{f}\left(t+\Delta t\right)-N_{f}\left(t\right)}{N_{g}\left(t\right)\Delta t}=\lambda$$

where *N_f_* is the number of failed items of an experiment, *N_g_* is the number of survived items, and Δt is the time infinitesimal.

### 3.1. Continuous Mode Reliability Simulation

**Figure 5:**
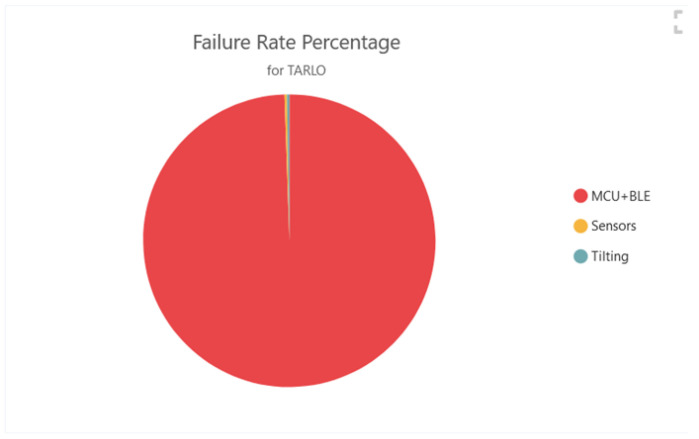
Failure rate percentage of each individual module composing the sensor node structure.

Regarding the ‘continuous’ mode, simulation results with 95% confidence bounds are reported in Table 1, where the MTBF of the system is expressed in hours, and the corresponding failure rate is evaluated, indicating the influence of each subsystem to the overall MTBF contribution. Such information is reported as well in the pie diagram of Figure 5, which expresses the weight of each subsystem on the overall failure rate. As could be imagined, due to the high number of components contributing to the MCU + BLE structure, most failures are expected in such a section.

To make the analysis more exploitable in the actual environment, we considered analyzing the case where a mission with 20% in a ground benign (GB) environment and 80% in a ground fixed (GF) environment could be considered. This inclusion was performed in order to take into account actual applications where the system could be moved or used in an environment that is of practical interest for the involved measurements. The results in the last column of Table 2 show how the environmental changes affect the overall system MTBF.

From the results reported in the table, it can be seen that the reliability of the whole sensor node is, in any case, determined by the MCU + BLE unit and that even if operating the node in GF for 20% of time halves and the MTBF with respect to 100% in GB, the estimated value for MTBF is still really satisfactory for orders of magnitudes higher than critical values for the considered application.

### 3.2. On Demand Mode Reliability Simulation

In the ‘on demand’ mode, two phases can be identified: the IDLE and the METERING. As described in the previous sections, during the IDLE, the MCU + BLE section is working 100%; the processor is active, as is the radio section, which is integrated into the processor, and data transmission occurs every 10 s. The tilting and vibration module is 1% active; the accelerometer is interrogated for 100 ms every 10 s to provide the data to the micro for the transmission, which takes place every 10 s. Finally, the sensors module is 1% active, which means that the BME 280 sensor is interrogated for 100 ms every 10 s to provide the data to the micro for transmission, which takes place every 10 s. Under these conditions, the simulations were carried out with the results shown in Table 3.

This working mode grants very large values for the MTBF of the tilting and vibration and sensors modules, nevertheless being the MCU + BLE module and the unit that mainly determines the node reliability, the resulting system performance is similar to those found in the previous section.

During the METERING phase, the MCU + BLE is always on, and the tilting and vibration module is working 5% of the time, as well as the sensor module. More in detail, in the tilting and vibration module, the accelerometer is interrogated for 100 ms every 2 s to provide the data to the micro for the transmission that takes place every 2 s. Instead, for the sensors module, the BME 280 sensor is interrogated for 100 ms every 2 s to provide the data to the micro for transmission, which takes place every 2 s. The results of this working mode are reported in Table 4.

In this working mode, the MTBF of the tilting and vibration and sensors module reduces, but still, the overall system performance is excellent from the reliability point of view.

**Figure 6:**
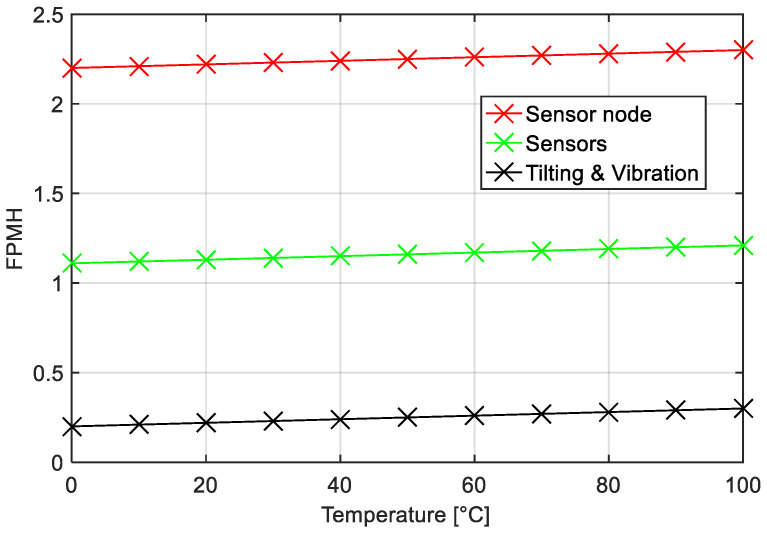
Expected number of failures for each individual subsystem composing the sensor node structure as a function of the temperature and considering the ‘continuous’ mode. Red line: failure modes of whole sensor node; light green line: failure rates of the sensors module; yellow line: failure mode of the tilting and vibration module, 100% GB.

The system was investigated for the ‘continuous’ mode and for the ‘on-demand’ mode, considering the METERING one, applying both temperature variations and working environment changes. Figure 6 shows the influence of these changes on the behavior of the whole system, expressed in failures per million hours (FPMH) and considering the node in ‘continuous’ mode. The red line shows the performance of the whole sensor node, whereas the light green line indicates that of the sensors module, and finally, the yellow line indicates that of the tilting and vibration module.

**Figure 7:**
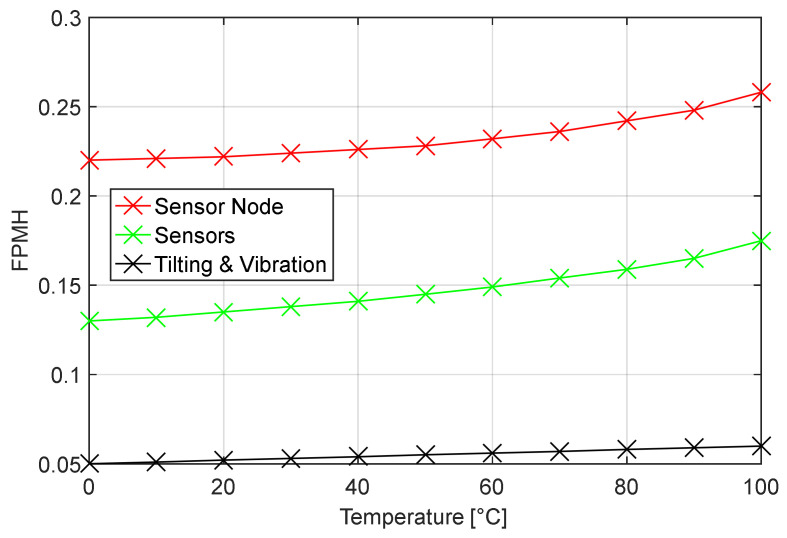
Expected number of failures for each individual subsystem composing the sensor node structure as a function of the temperature and considering the ‘on demand’ mode. Red line: failure modes of whole sensor node; light green line: failure rates of the sensors module; yellow line: failure mode of the Tilting and Vibration module in 100% GB.

Figure 7 reports the results obtained through the same analysis when applied to the sensor node in the METERING phase, and the results are displayed in Figure 6. It is evident that, as expected, the overall estimated FPMH obtained in this case is lower than the one obtained in the previous configuration; nevertheless, the sensors module FPMH, and consequently the whole sensor node failure rate, shows a higher temperature dependency. This can be explained by the fact that the warmup needed for the sensing part to properly acquire signals can induce in this module an additional stress according to the Arrhenius aging law.

**Figure 8:**
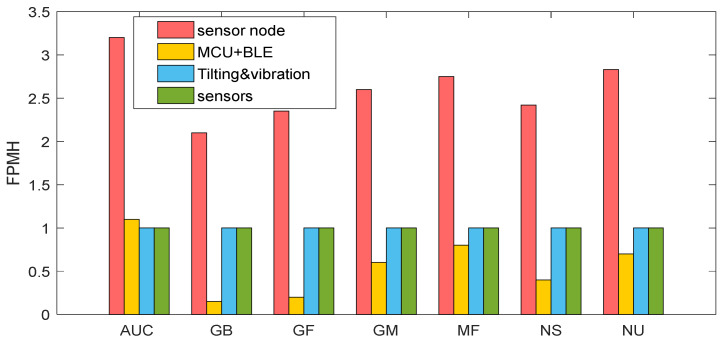
Expected number of failures for each individual subsystem (module) composing the sensor node in ‘continuous’ mode and considering the working environments previously listed. Red bars: failure rates of the whole sensor node; orange bars: failure rates of the MCU + BLE module; blue bars: failure rates of tilting and vibrations module; green bars: failure rates of the sensors module.

**Figure 9:**
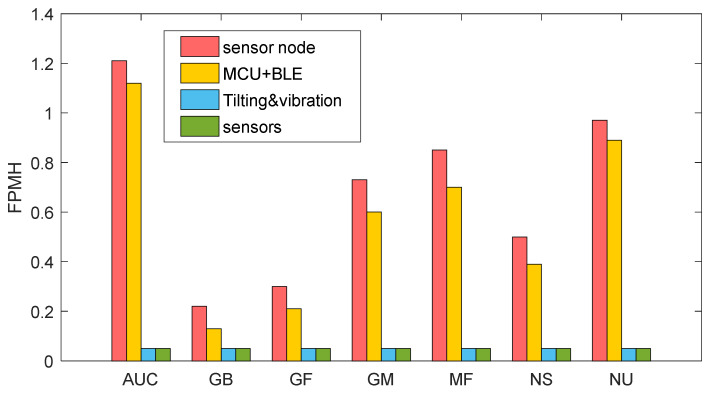
Expected number of failures for each individual subsystem (module) composing the sensor node under the ‘on demand’ METERING operating condition and considering the working environments previously listed. Red bars: failure rates of the whole sensor node; orange bars: failure rates of MCU + BLE module; blue bars: failure rates of tilting and vibrations module; green bars: failure rates of the sensors module.

To complete the life performance assessment of the proposed device, the analysis was conducted in different working environments, and the results are shown in Figure 8 and Figure 9. The working environments, without considering any dormant one, are the ground benign (GB), ground mobile (GF), (GM), missile flight (MF), naval sheltered (NS), naval unsheltered (NU), and airborne uninhabited cargo (AUC) proposed in an increasing degree of severity.

In Figure 8, the overall system failure rate (red bar) is the composition of the individual failure rates of each subsystem (module): MCU and BLE (orange), tilting and vibration (blue), and sensors (green). It is evident from the proposed analysis that the main difference between Figure 8 and Figure 9 is the marginal contribution of the sensing modules during METERING with respect to the ‘continuous’ mode, which is represented in Figure 8.

## 4. Measurement and Results

To experimentally assess the performance of the proposed sensor node, three different types of tests were carried out to exploit the climatic chamber (ACS DY 200). The first one aimed to evaluate the resolution of the sensor node in terms of tilt in realistic conditions. The second one was devoted to testing the tilting measurement performance, and finally, the last one was designed to evaluate the stability of the sensing devices at different operating temperatures.

### 4.1. Tilting Measurement Performance Assessment

**Figure 10:**
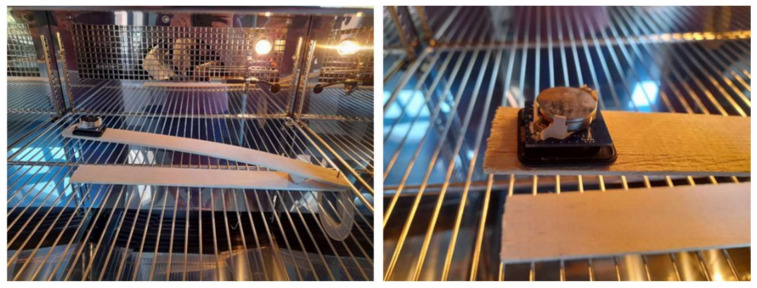
Sensor mounted on the cantilever in the climatic chamber.

To validate the ability of the sensor node to measure small tilting variations in variable climatic conditions and to emulate the monitoring of fine timber movements, an accelerated test was carried out using a climatic chamber. The sensor was mounted on a spruce wood cantilever. The cantilevered was designed to have a fast mechanical response to the variations of climatic conditions in such a way that all the possible deformations of the wood (longitudinal along the fibers, transverse and tangential) could produce an angle variation that could be sensed by the sensor node. The setup can be seen in Figure 10.

The sensor was fixed at the cantilever-free end; to reduce the effect of the temperature in the sensor responses, the test was carried out at a fixed temperature of 40 °C, which can be assumed to be the upper bound for the temperature in typical sensor node installation environments. In this condition, the humidity was reduced from 90 %RH to 25 %RH in 1 h. Data were acquired for a 15 h time window. The humidity varied in the middle of the time window.

**Figure 11:**
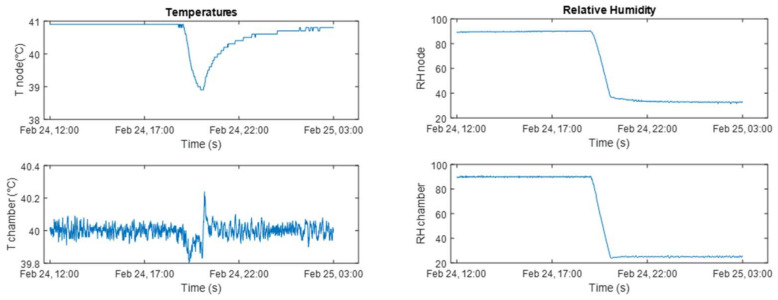
Comparison between the temperature and the relative humidity measured by the climatic chamber embedded sensors (Tchamber, and RH chamber) and by the sensor node (Tnode and RH node).

Figure 11 shows the comparison between the temperature and the relative humidity measured by the built-in sensor of the climatic chamber and by the sensor node. The difference between the two measured temperatures is provided by the different positions of the sensors in the chamber. Moreover, the chamber temperature sensor was also exploited by the chamber control system and, thus, can be considered a reference. Concerning the relative humidity, the difference between the measurements, during the constant phase at 25% RH is provided by the different positions of the node with respect to the chamber humidity sensor.

**Figure 12:**
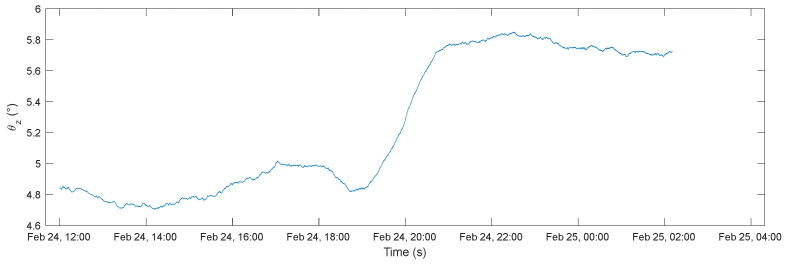
Angle $\phi_{z}$ estimated from the accelerometer output and exploiting Equation (1) during the test.

Figure 12 represents the angle $\phi_{z}$ derived from the acceleration measurements as described in Equation (1). From the graph, it can be noted that the proposed sensor node is able to detect small variations in the tilting angles (smaller than 1°); moreover, the detected trend is coherent with the expected movements of the cantilever end due to the deformation related to the variation in the wood water content. In fact, from what can be seen from the measurements, the spruce cantilever end moved upward during the test.

The experimental results obtained in this test show the ability of the device to detect small tilting variations in a structure provided by a change in the environmental parameters. In this context, the device can be permanently mounted in a critical part of a wooden structure, such as a pillar or a cantilever in the case of a pulpit or an Ambon, for instance, or in an artwork frame to detect tilting variations in the long run. Assuming that small movements in wooden structures, related to moisture variations, for example, can be considered normal within certain limits, the tilting data acquired by the device can be used to monitor abnormal movements which are related to permanent changes in the structure (e.g., incipient cracks in pillars or frames).

### 4.2. Performance Assessment of the Measurment Devices in Temperature

The performance of the proposed node was tested in situations emulating possible application scenarios. To clarify the aim of the test described in this subsection, we refer to a typical application, in which the device can be mounted, for example, on a pillar to monitor the movement of the pillar itself in the long run. In this case, the sensor must be able to provide a data history containing information about the very small movements of the structure so as detect if there are permanent changes in the structure given, for example, by incipient cracks.

The performance of the sensors embedded in the node must be guaranteed to be stable in the temperature working range. In particular, possible scenarios for the node installation can have temperatures ranging from 5 °C, for example, in old churches in winter, to 50 °C maximum in hot environments.

To assess the performance of the sensing devices embedded in the node as a function of temperature and humidity variations, the sensor node was tested in the climatic chamber in a temperature interval ranging from 5 °C to 50 °C.

In particular, both static tests and dynamic tests were conducted on the sensor node by varying the temperature and humidity.

#### 4.2.1. Static Tests

Static tests were performed to verify the influence of the temperature and humidity variations on the sensor’s output offsets and noise. Since the climatic chamber compressor transmits vibrations to the sensor node, to quantify the influence of the temperature on the accelerometer (vibration and tilt module) output, two separate tests were performed, in which the climatic chamber was initially set at temperatures of 5 °C and 50 °C, respectively, then in both cases the chamber was turned off, and the free responses toward the environmental conditions were exploited to evaluate the relationship between the temperature and the accelerometer output offsets and noises.

To test the BME280 (sensors module), the temperature was varied according to a time profile consisting of steps that started from 5 °C and arrived at 50 °C, with a final step at 25 °C. Each step lasted 2 h to ensure that a steady state was reached. Transitions between the different temperatures lasted 30 min. RH was kept constant during the test with a value of 50%.

**Figure 13:**
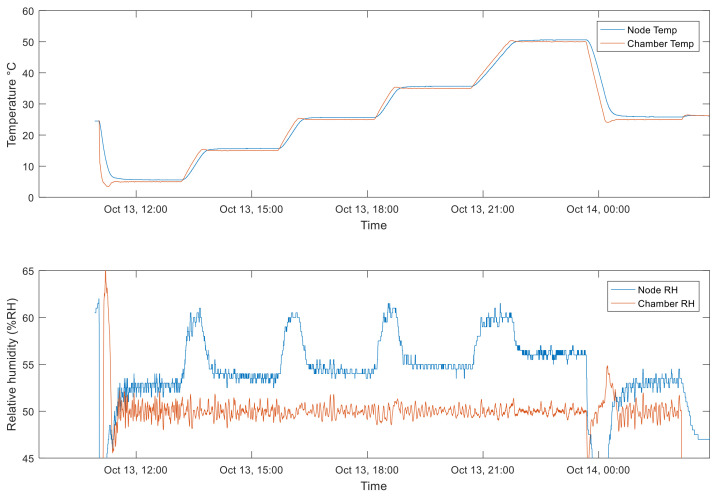
Environmental conditions for the static tests of BME280. Red lines in both subplots show the temperature and RH measured by the built-in sensors of the chamber, whereas blue lines show the measurements of the same quantities performed by the tested sensor node.

The results obtained with the above-described temperature profile are shown in Figure 13 in terms of a comparison between the responses of the node BME280 sensor and the ones provided by the climatic chamber built-in sensors (used as references). As can be noticed, the humidity measured by the node had a small offset with respect to the one measured by the climatic chamber. This is due to the different positions of the node with respect to the climatic chamber sensor. The spikes are instead related to the temperature variations during the transients. 

**Figure 14:**
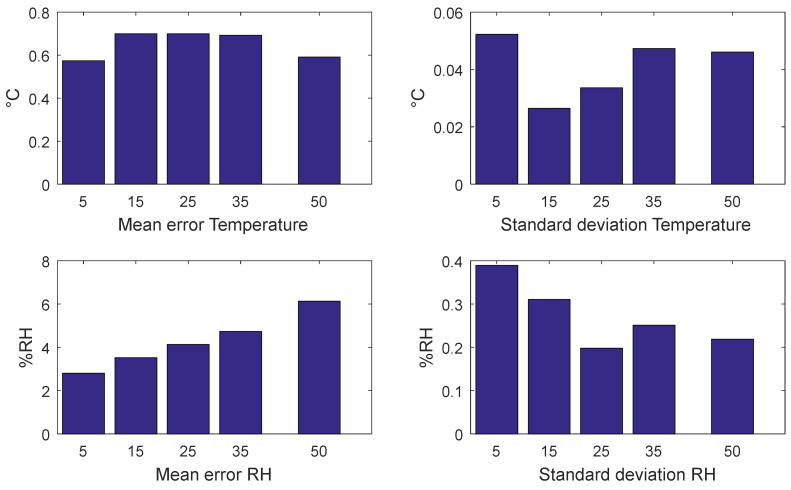
Mean error and standard deviation between the temperature and relative humidity measured by the node and the reference during the steady states.

Figure 14 shows the mean error and the standard deviation between the measured environmental parameters and the reference by the climatic chamber. It can be noted that, at a steady state, the error in temperature was less than 0.8 °C, while for the relative humidity, there was a little offset due to the different positions between the reference sensor and the node; however, the error is lower than 6%RH at the temperature of 50 °C.

**Figure 15:**
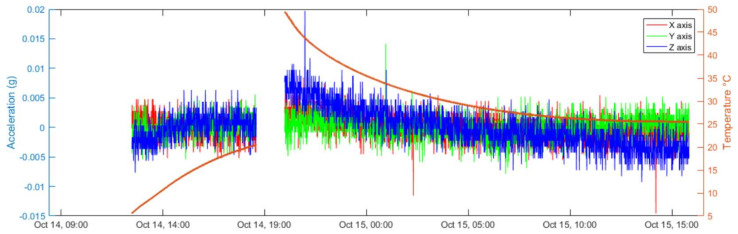
Values of the vibration measurements along the three axes during transient temperature variations, starting from 5 °C and 50 °C, respectively, in the absence of external vibrations. Average values are obtained over mobile window 16 min-long.

Figure 15 shows the results obtained during the transient responses of the climatic chamber (chamber turned off, temperature from 5 °C to 20 °C, and from 50 °C to 20 °C), as described in the previous subsection. In particular, the figure shows the behaviour of the accelerometer output signals. Here, the response of the three axes as a function of time is represented, together with the chamber temperature (red solid line in the figure). A dependency on the temperature can be noticed, in particular, as the offset increases with the temperature for all axes.

**Figure 16:**
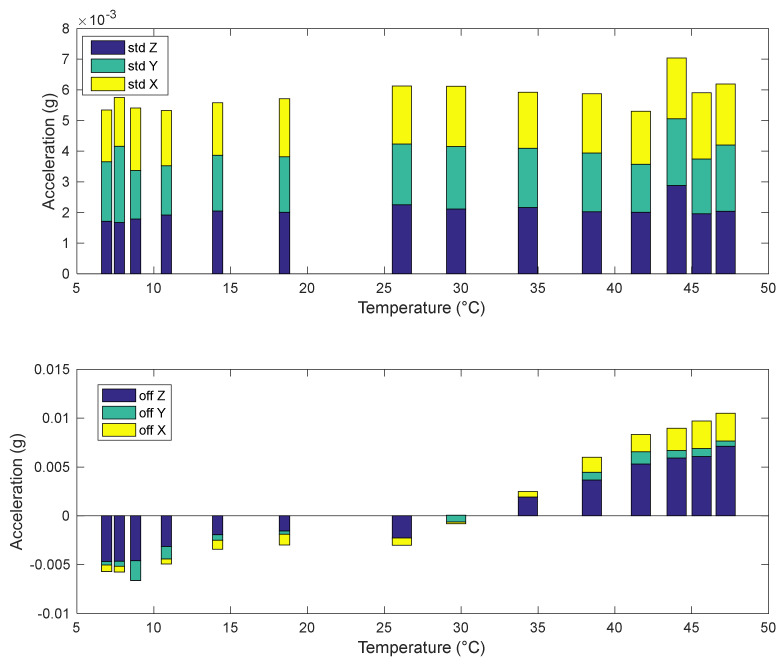
Standard deviation (**top**) and offset (**down**) behaviour as a function of temperature for the accelerometer outputs (axes). Standard deviations and offsets (**down**) are evaluated over mobile windows of about 16 min.

This dependency, together with the dependence of noise on temperature, is clarified in Figure 16, which shows the behaviour of the average values (offsets) and of the standard deviations (noise root mean square value) of the accelerometer outputs as a function of the temperature. The average and standard deviations are evaluated over mobile windows about 20 min long. It can be noticed that the standard deviations remain almost constant over the tested temperature range, while the offsets, as previously stated, increase with temperature. Moreover, the three axes behave in the same way over the whole temperature range as far as noise is concerned, whereas a higher offset characterizes the z-axis signal (which is generated by the off-plane structure of the MEMS).

#### 4.2.2. Dynamic Tests

Dynamic tests were carried out by placing inside the climatic chamber a stepper motor that rotates the sensor node at a constant speed (1 rotation every 1036 s).

During the tests, the temperature profile described in Section 4.2.1 was used.

**Figure 17:**
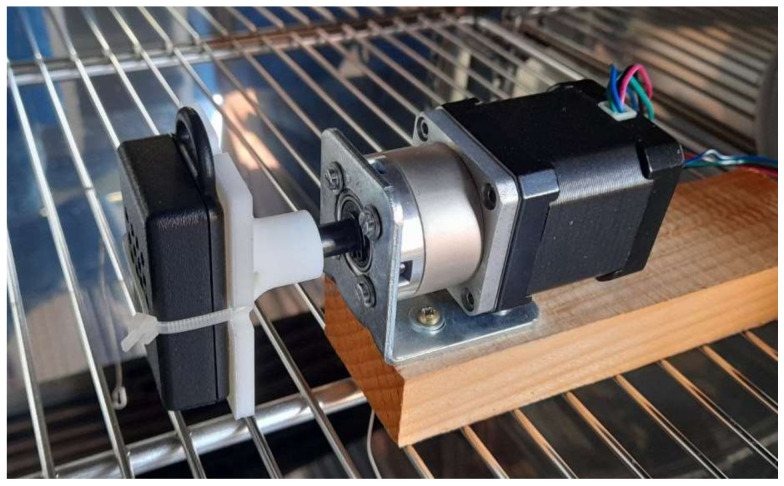
Sensor node mounted on the stepper motor in the climatic chamber.

Figure 17 shows the arrangement of the node and the motor in the chamber. The node rotated around the z axis of the accelerometer reference system, which was parallel to the motor axis.

The purpose of the test was to assess the sensor node performance in dynamic conditions and in different environmental conditions.

**Figure 18:**
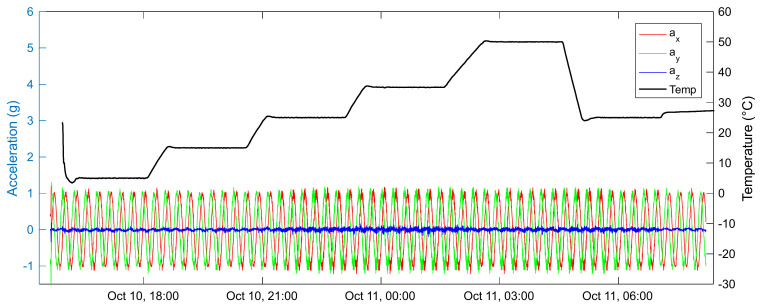
Acceleration measurements during the test; red, green, and blue lines indicate *a_x_, a_y_,* and *a_z_*, respectively. The black line indicates temperature.

Figure 18 presents the behaviour of the acceleration components, *a_x_, a_y_,* and *a_z,_* measured by the node during the test at different temperatures. By performing the zero-crossing of the acceleration signals, the length of the periods was estimated, considering the data acquired from the X and Y axes of the accelerometer.

For both the X and Y axis, the estimated period shows no appreciable error related to temperature variations.

## 5. Conclusions

Pointwise environmental monitoring in museums near artworks and in cultural places is nowadays quite common. Nevertheless, tilting and vibrations in artworks and wooden structures are rarely monitored due to the cost of sensing devices. In this work, a low-cost, low-power sensor node was described that was able to combine environmental monitoring with the detection of motion, tilt, and vibrations which is especially useful for the monitoring of artworks during transportation in supply chain visibility. Due to careful electronic design, the expected battery life is about 1.6 years. Detailed reliability analysis was conducted by considering different application scenarios and conditions. This study demonstrated the suitability of the proposed node for the implementation of monitoring systems with very long availability, especially considering the optimization of the sensor node life due to the operating mode used for shock and movement detections. Some experimental tests performed in different environmental conditions were used to assess the device’s performance in terms of vibration, angle measurements, and reliability.

## Figures and Tables

**Table 1:** Reliability analysis of the sensor node structure considering all the blocks working 100% of the mission for continuous operating mode in GB and 25 °C.

Node/ Module Name	Failure Rate λ [f/10^6^ h]	MTBF [h]	Failure Rate λ [%]	Failure Rate, λ [f/10^6^ h] Active	Failure Rate, λ [f/10^6^ h] Dormant	Failure Rate λ [f/10^6^ h] Mean
Sensor Node	0.122896	8,136,956.03	100.00	0.122896	6.293960 × 10^−4^	0.122896
MCU + BLE	0.122231	8,181,252.62	99.46	0.122231	5.628551 × 10^−4^	0.122231
Tilting and vibrations	3.327044 × 10^−4^	3.01 × 10^9^	0.27	3.327044 × 10^−4^	3.327044 × 10^−5^	3.327044 × 10^−4^
Sensors	3.327044 × 10^−4^	3.01 × 10^9^	0.27	3.327044 × 10^−4^	3.327044 × 10^−5^	3.327044 × 10^−4^

**Table 2:** Reliability analysis of the sensor node structure considering all the blocks working on continuous mode and considering 20% in GB and 80% in GF environments, in comparison with mission in 100% GB at 25 °C.

Node/ Module Name	Failure Rate λ [f/10^6^ h] Mission 100% GB	MTBF [h] Mission 100% GB	Failure Rate λ [f/10^6^ h] Mission 20% GB + 80% GF	MTBF [h], Mission 20% GB + 80% GF
Sensor node	0.122896	8,136,956.03	0.247561	4,039,411
MCU + BLE	0.122231	8,181,252.62	0.237165	4,216,480
Tilting and vibrations	3.327044 × 10^−4^	3.01 × 10^9^	0.005198	192,377,682
Sensors	3.327044 × 10^−4^	3.01 × 10^9^	0.005198	192,377,682

**Table 3:** Reliability analysis of the sensor node structure considering all the blocks working on demand mode and the device in IDLE mode, in 100% GB at 25 °C.

Node/ Module Name	Failure Rate λ [f/10^6^ h]	MTBF [h]	Failure Rate λ [%]	Failure Rate, λ [f/10^6^ h] Mean
Sensor Node	0.122237	8,180,807.27	100.00	0.122898
MCU + BLE	0.122231	8,181,252.62	99.99	0.122232
Tilting and vibrations	3.327044 × 10^−6^	3.01 × 10^11^	2.72 × 10^−3^	3.33 × 10^−4^
Sensors	3.327044 × 10^−6^	3.01 × 10^11^	2.72 × 10^−3^	3.33 × 10^−4^

**Table 4:** Reliability analysis of the sensor node structure considering all the blocks working on demand mode with the device in METERING mode, in 100% GB at 25 °C.

Node/ Module Name	Failure Rate λ [f/10^6^ h]	MTBF [h]	Failure Rate λ [%]	Failure Rate, λ [f/10^6^ h] Mean
Sensor Node	0.122264	8,179,026.34	100.00	0.122896
MCU + BLE	0.122231	8,181,252.62	99.97	0.122231
Tilting and vibrations	1.663522 × 10^−5^	6.01 × 10^10^	1.36 × 10^−2^	3.33 × 10^−4^
Sensors	1.663522 × 10^−5^	6.01 × 10^10^	1.36 × 10^−2^	3.33 × 10^−4^
